# Current and Potential Applications of Green Membranes with Nanocellulose

**DOI:** 10.3390/membranes13080694

**Published:** 2023-07-25

**Authors:** Stefanos (Steve) Nitodas, Meredith Skehan, Henry Liu, Raj Shah

**Affiliations:** 1 Department of Materials Science and Chemical Engineering, Stony Brook University, Stony Brook, NY 11794, USA; meredith.skehan@stonybrook.edu (M.S.); henry.liu@stonybrook.edu (H.L.); 2 Koehler Instrument Company Inc., Bohemia, NY 11794, USA; rshah@koehlerinstrument.com

**Keywords:** nanocellulose, membranes, water treatment, wastewater, gas separation, bacterial nanocellulose

## Abstract

Large-scale applications of nanotechnology have been extensively studied within the last decade. By exploiting certain advantageous properties of nanomaterials, multifunctional products can be manufactured that can contribute to the improvement of everyday life. In recent years, one such material has been nanocellulose. Nanocellulose (NC) is a naturally occurring nanomaterial and a high-performance additive extracted from plant fibers. This sustainable material is characterized by a unique combination of exceptional properties, including high tensile strength, biocompatibility, and electrical conductivity. In recent studies, these unique properties of nanocellulose have been analyzed and applied to processes related to membrane technology. This article provides a review of recent synthesis methods and characterization of nanocellulose-based membranes, followed by a study of their applications on a larger scale. The article reviews successful case studies of the incorporation of nanocellulose in different types of membrane materials, as well as their utilization in water purification, desalination, gas separations/gas barriers, and antimicrobial applications, in an effort to provide an enhanced comprehension of their capabilities in commercial products.

## 1. Introduction

Research within the fields of material science and nanotechnology has been on a steady rise over the past several years. By isolating and modifying certain advantageous properties of newly developed materials, such as nanostructures, the latter can be applied to optimized processes to facilitate the improvement of several aspects of our lives. Given the unique size of nanomaterials, nanostructured materials can be modified at the scale of 1–100 nm, resulting in the improvement of a variety of properties within products used in everyday life. In recent years, one such material has been nanocellulose. Nanocellulose is a naturally occurring nanomaterial extracted from the cell walls of plant fibers, plant biomass, and algae [1,2]. This green nanomaterial can be obtained either as nanocrystalline/nanofiber cellulose via top-down biosynthesis by disintegration of plant materials or as bacterial nanocellulose through bottom-up biosynthesis [3,4,5].

Nanocellulose is characterized by chemical inertness, high tensile strength, and biocompatibility; in addition, it exhibits dimensional stability, a low coefficient of thermal expansion, and the ability to modify its surface chemistry [6,7,8,9,10]. In recent studies, these unique properties of nanocellulose have been enhanced and applied to processes in the areas of water purification [11], automotive [12], food [1,13], and membrane separations [14]. The industry prioritizes the low cost and efficient production involved in utilizing nanocellulose as an alternative to conventional cellulose. Compared to traditional cellulose, nanocellulose has a higher surface area, aspect ratio, and Young’s modulus, allowing its application to adsorption, photocatalysis, flocculation, and membranes [15]. In addition, the research interest in nanocellulose-based materials for environmental applications is rapidly growing due to increasing environmental problems, water contamination, and the severe risk of oil pollution [16,17].

Regarding water purification, it has been shown that modified variants of nanocellulose reduce the concentration of contaminants in wastewater. For instance, carboxymethyl nanocellulose stabilized nano zero-valent iron has been proven to be effective in reducing the presence of hexavalent chromium within wastewater [11]. Nanocellulose has been the subject of a wide spectrum of research efforts for its utilization in membranes for multifunctional wastewater treatment and adsorption, with a primary focus on improving the permeability of nanocellulose and extracting purified liquid [18,19,20,21].

Nanocellulose has also been applied to gas separation processes such as regulating membranes [22,23]. These nanocellulose membranes allow for the capture and storage of certain gases, including CO_2_, which is prevalent in many industrial processes involving power generation [24,25]. This action decreases carbon emissions, leading to lower greenhouse gas pollution. Nanocellulose membranes have also been applied to biomedical devices. This provides greater precision in chemical regulation given its dynamic permeability, which is optimal for medical environments [26]. The current study constitutes a comprehensive review of the aforementioned important applications of nanocellulose-based membranes, with emphasis on the environmental sector (water purification, gas separations).

## 2. Types of Nanocellulose

Over the past few years, nanocellulose has emerged as one of the most promising materials for a variety of applications, largely due to its biodegradable nature. A highlighted advantage of nanocellulose is also its diverse size/dimensions which enable its versatility across multiple fields. With pollution on the rise and environmental issues greater than ever before, this material may pose solutions to issues in this specific area. The market for nanocellulose has been on a steady increase, with estimates of it being worth $660 million by the end of 2023 [27] and $783 million by 2025 [28]. Paper and pulp products represent the majority of the nanocellulose market; nanocellulose has been used as an additive in papermaking to produce lighter paper that exhibits enhanced properties, such as higher printing quality, improved mechanical strength, and less transparency [28,29]. Similar trends have been observed with patents associated with cellulose nano-objects; from 2010 to 2017, about 4500 patents referring to nanocellulose were published [30].

Currently, nanocellulose can be produced in many different forms, including one-dimensional (nanofibers/microparticles), two-dimensional (films), and three-dimensional (hydrogels/aerogels) variants. There are three main types of nanocellulose: cellulose nanocrystals (CNC), cellulose fibers (CNF), and bacterial nanocellulose (BNC) [31]. Cellulose nanocrystals (CNC) are a crystalline derivative of nanocellulose extracted through strong acid hydrolysis at high temperatures [32]. CNCs possess high thresholds in aspect ratio, surface area, and mechanical strength, which renders them ideal for applications on surfaces that require reinforcement.

Cellulose fibers (CNF) are microfibrils separated from nanocellulose and obtained by breaking down complex nanocellulose structures through chemicals and mechanical means. CNFs possess high plasticity regarding the dimensions of the material. Depending on the plant source of the nanocellulose, CNFs can have varying width and diameter ranges, rendering them ideal for applications that require flexibility in the size of the applied nanomaterials.

Bacterial nanocellulose (BNC or BC) is a promising natural biopolymer that can be produced by specific bacterial species, such as an exopolysaccharide of β-D glucopyranose [33]. It can be obtained through cultivation in a bacterial environment saturated with glucose, phosphate, and oxygen [34]. The versatile nature of this form of nanocellulose has generated greater research related to biomedical applications. BNC exhibits very good mechanical properties, whereas its nanostructured morphology and water-holding capacity render bacterial nanocellulose an ideal material for cellular immobilization and adhesion [35,36]. The properties of the three types of nanocellulose are compared in Table 1, where it can be seen that both CNC and BNC are characterized by high crystallinity, while CNC and CNF exhibit high Young’s Modules values [31].

## 3. Applications of Nanocellulose-Based Membranes

### 3.1. Desalination and Wastewater Treatment

About 4 billion people are experiencing water scarcity at least one month per year, whereas 1.8 billion people are facing an absolute water shortage [37]. It has also been reported that more than 63 million Americans have been exposed to more water contamination in recent decades [38]. Membrane-based desalination offers one solution to the water scarcity issue, as it supplements the natural hydrological cycle with freshwater obtained from seawater and other non-potable sources [39]. Additionally, membrane-based desalination is more cost-effective than traditional chemical treatments. Nanocellulose in particular has potential in membrane-based desalination because its unique properties lend themselves to a more efficient and eco-friendly membrane [39]. Nanocellulose exhibits high surface area and high tensile strength, and because it is naturally derived, it is non-toxic to humans and the environment [40].

Membrane-based desalination can be accomplished through various methods, including nanofiltration, reverse osmosis, pervaporation, ultrafiltration, and distillation. Nanocellulose has been successfully employed in all aforementioned methods, with different forms of nanocellulose displaying varying efficiency in each method [41,42,43]. Cellulose nanocrystals (CNCs) have been found to have applications in nanofiltration, reverse osmosis, and pervaporation. Yang et al. found that polydopamine-modified CNCs have significant applications in nanofiltration. When deposited on a thin-film nanocomposite membrane, the addition of the modified CNC produced pure water permeability of 128.4 L m^−2^ h^−1^ (LMH)/bar, Congo red rejection of 99.91%, and salt permeation of 99.33% [44]. Comparatively, cellulose nanofibrils (CNFs) have been found to have applications in ultrafiltration as well as nanofiltration and reverse osmosis.

**Figure 1:**
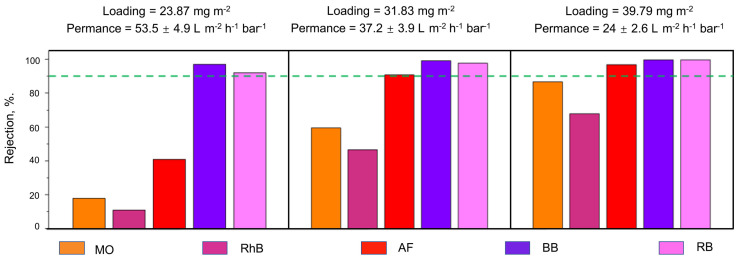
Comparison of pure water permeance and rejection of rGO:CNF_(1:1)_ membrane for various loading after 3 min plasma treatment (Adapted from Mohammed et al. [45]).

Mohammed et al. carried out a study to test the performance of reduced graphene oxide/cellulose nanofibrils (CNFs) in membranes used for nanofiltration [45]. The study found that reduced graphene (rGO) membranes with CNF exhibited a pure water permeance of 37.2 LMH/bar, whereas rGO membranes without CNF had a reduced pure water permeance of 0.33 L m^−1^ h^−1^ bar^−1^. This result can be seen in Figure 1. The figure shows the varying pure water permeance of three rGO membranes for five different organic dyes. The membranes vary in their rGO loading but have a fixed 1:1 ratio of rGO to CNF. Additionally, all three membranes received three minutes of oxygen plasma etching treatment in order to increase the number of nanopores on the surface of the membrane. The dyes tested include methyl orange (MO), rhodamine B (RhB), acid fuchsin (AF), brilliant blue (BB), and rose bengal (RB). As shown in the figure, the membrane with a 23.87 mg m^−2^ loading had the highest pure water permeance but only had more than 90% rejection for ⅖ of the dyes. The membrane with a 39.79 mg m^−2^ loading displayed improved rejection but suffered from a lower pure water permeance. In addition, it was still not able to obtain 90% rejection for MO and RhB. Consequently, the membrane with a 31.83 mg m^−2^ loading was determined to be the most well-rounded membrane due to its ability to reject more than 90% of the AF, RB, and BB dyes while maintaining an acceptable pure water permeance [45].

Other research studies with nanocellulose-based materials have focused on the removal of heavy metals from wastewater. Heavy metals can stem from the effluents of several industries, including electroplating, metallurgical processes, and mining [46]. These activities constitute hazards to the environment and humans. For effective heavy metal removal by adsorption processes, the surface chemistry of the nanocellulose-based membrane must be tailored for the removal of specific metal species [47]. As an example, negatively charged carboxylated CNF coupled with trimethylolpropane-tris-(2-methyl-1-aziridine) propionate and graphene oxide was found to be an excellent adsorbent of numerous cations of heavy metals, including Pb^2+^, Cd^2+^, and Cu^2+^ [48]. Several surface modifications of nanocellulose have been investigated, with carboxylation being the most studied method for enhancing the sorption capacity of nanocellulose [49]. Tetramethylpiperidine-1-oxyl (TEMPO)-oxidized nanocellulose adsorbents present outstanding adsorption capabilities for divalent cations [50]. The carboxyl group provides a strong negative charge to nanocellulose, which permits the adsorption of even radioactive species [47].

**Figure 2:**
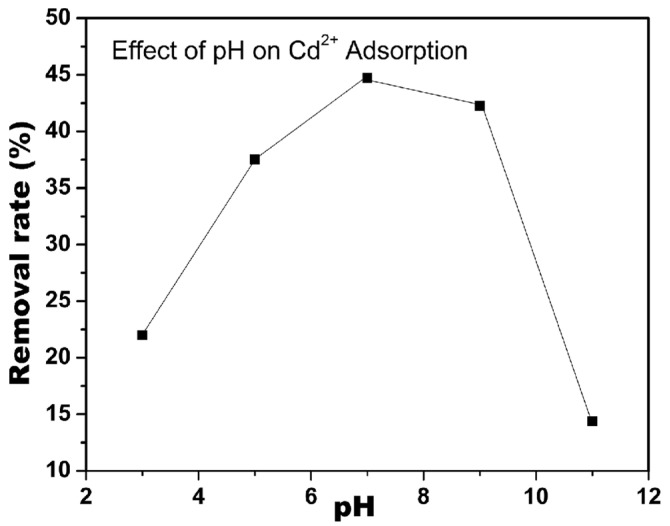
Effect of pH value on the Cd^2+^ adsorption efficiency of CNF (Adapted from Sharma et al. [51]).

In addition to the surface modification of the nanocellulose surface, the pH of the solution is critical for the selectivity of nanocellulose over heavy metal ions. Sharma et al. reported that the adsorption of Cd^2+^ from cadmium(II) nitrate solution using CNF modified by the nitro-oxidation method was optimum at pH 7 and decreased in acidic and basic conditions, as can be seen in Figure 2 [51]. At low pH values, some carboxylic acid groups became neutral, resulting in weaker electrostatic interactions overall. At high pH values, on the other hand, some CNF nanofibers were denatured, and consequently, the Cd^2+^ adsorption capacity of the system decreased.

Bacterial nanocellulose can also be employed for treating water contamination [52,53]. This material covers a smaller range of applications than CNC and CNF, with one of them being membrane distillation. Wu et al. produced a membrane for photothermal membrane distillation composed of polydopamine (PDA) particles and bacterial nanocellulose arranged in a bilateral composition and exposed to (tridecafluoro-1,1,2,2-tetrahydrooctyl)-trichlorosilane (FTCS) vapor [54]. They found that the membrane exhibited a salt rejection greater than 99.9%, a solar energy-to-collected water efficiency of 68%, and a 1.0 kg m^−2^ h^−1^ permeate flux. Additionally, the membrane allowed for ease of cleaning due to its ability to partially disinfect itself when exposed to sunlight.

**Figure 3:**
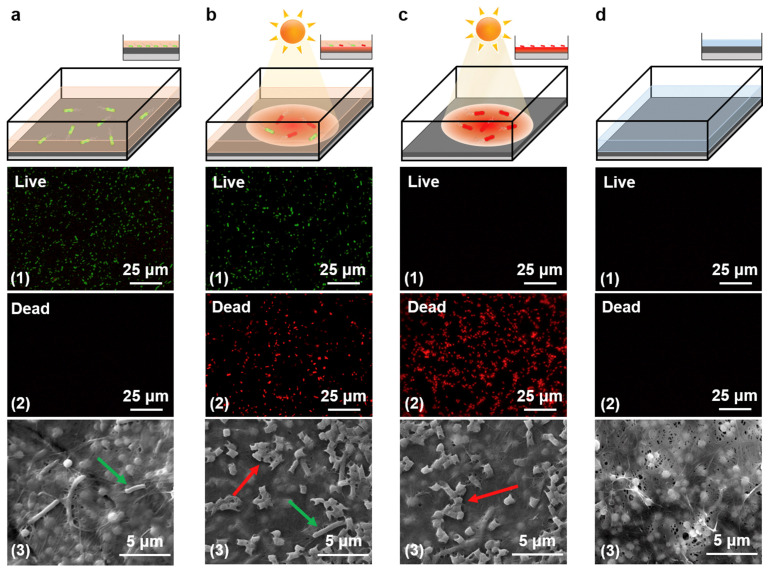
Activity measurements of photothermal disinfection. Schematic (1), fluorescence (2), and SEM images (3) of (**a**) FTCS-PDA/BNC membrane after exposure to water contaminated with *E. coli* for 1 h, (**b**) FTCS-PDA/BNC membrane after in situ PMD operation for 1 h with water contaminated with *E. coli*, (**c**) FTCS-PDA/BNC membrane after the water contaminated with *E. coli* was drained from the top surface, and exposure of the membrane to solar light (1 kW m^−2^) for a duration of 10 min, (**d**) FTCS-PDA/BNC membrane after exposure to light and washing using distilled water (Adapted from Wu et al. [54]).

Figure 3 shows how effective light irradiation has been at eliminating bacteria close to the FTCS-PDA/BNC membrane surface [54]. Fluorescent staining was used to color-tag dead and live cells red and green, respectively. Scanning electron microscope (SEM) imaging was used to observe the membrane surface, with red and green arrows used to indicate dead and live cells. In Figure 3a, which represents the control experiment, a solution of more than 324 live *E. coli* cells/mL was used to simulate bacteria-contaminated water. Under dark conditions and after 1 h, the fluorescence imaging shows the presence of live *E. coli* cells as well as the absence of dead cells. In Figure 3b, light irradiation (1 kW m^−2^) was applied in addition to the *E. coli* solution to simulate in situ conditions. After 1 h, the presence of live and dead cells can be observed in Figure 3(b1–b3). Figure 3c depicts the membrane surface after the feed water from Figure 3b was drained and the membrane was exposed to light irradiation (1 kW m^−2^) for a duration of 10 min. After this second dose of light irradiation, the membrane only exhibited dead cells, which suggests the rise in membrane temperature (~78 °C) in addition to the removal of the bulk top water was successful in killing the *E. coli.* Finally, Figure 3d depicts the membrane surface after being washed in DI water for 5 min. As seen in Figure 3(d1–d3), no *E. coli* cells were detected. This is a significant observation because it suggests that the membrane can be effectively cleaned without having to depend on invasive methods that can change the chemical composition or integrity of the membrane.

Another variant of nanocellulose, nanocellulose acetate (NCA), also displayed promising desalination applications and anti-biofouling properties. In a study conducted by Morsy et al., NCA was prepared from rice straw waste by acidic hydrolysis, and the NCA was incorporated into reverse osmosis membranes via phase inversion [55]. The NCA membranes displayed decreased relative protein adsorption compared to the pristine membranes and increased water flux and salt rejection [55]. The aforementioned studies illustrate how the use of CNC, CNF, or BNC can differ according to which desalination method is preferred. However, as all of the prior materials are derived from nanocellulose, the latter can be considered a widely applicable material for desalination processes.

Another application of nanocellulose that has received attention in the last few years is in solar evaporators for producing freshwater from seawater. Solar evaporation is an attractive technology that combines water and solar energy. It has enabled an array of emerging applications, including contaminated water purification, seawater desalination, electric generation, steam sterilization, and fuel production, especially in resource-limited regions and countries [56]. Compared to many synthetic polymer-based evaporators, nanocellulose-based evaporators are expected to benefit from the NC’s abundant reserves and renewable features [57]. In the study of Wu et al., it is shown that the microporous network of cellulose composite-based evaporators results in performance improvements such as high evaporation rates and salt resistance [57].

In another work by Jian et al., a flexible, scalable, and biodegradable photothermal bilayered evaporator for highly efficient solar steam generation was demonstrated [58]. The bilayered evaporator consisted of BNC loaded with a high concentration of polydopamine (PDA) particles during its growth. The size of the PDA particles was tailored to achieve light absorption properties matching the solar spectrum. This hybrid biodegradable material introduced in the evaporator exhibited good photothermal conversion and heat localization, leading to a high solar steam generation efficiency of 78%, thus showing a promising approach to tackle the global water crisis.

Table 2 summarizes the findings of the research works studied in this section.

### 3.2. Gas Separation

A prominent application of nanocellulose is in gas separation technologies, with particular application in carbon capture [59,60,61,62]. Currently, carbon dioxide (CO_2_) accounts for about 76% of total greenhouse gas emissions, and this number is only expected to rise, according to the U.S. Energy Information Administration [63]. As such, carbon capture and storage may play an important role in tackling this issue. Carbon capture can be broken into three strategies, including pre-combustion capture, oxyfuel processes, and post-combustion capture [63]. Among these strategies, post-combustion techniques hold particular interest in most carbon capture and storage (CSS) projects because incorporating different CO_2_ separation technologies will not disturb existing processes [63].

Basic CO_2_ gas separation methods common in CSS are adsorption and membrane separation, which can be realized through gas separation membranes [63]. Materials used for these membranes must be abundant, low-cost, and sustainable, and the membranes themselves must exhibit high CO_2_ permeability and selectivity to be successful. Nanocellulose meets all these requirements, as cellulose can be produced at more than 100 million tons per year, and nanocellulose itself was found to be biodegradable and cheap at $2 USD per kg in 2011 [63,64,65]. Lastly, nanocellulose has properties that lend themselves to stronger and more effective membranes and adsorbents, such as a surface area ranging from 100 to 200 g/m^2^, a tensile strength ranging from 7.5 to 7.77 GPA, and a Young’s Modulus of 110–220 GPa [66].

Nanocellulose membranes can be created using a variety of methods, including vacuum filtration, solvent casting, dip coating, and electrospinning [67]. Without any modification, nanocellulose membranes exhibit low gas permeability [68]. As a result, they can be employed as gas barrier materials instead of gas permeation materials. On the other hand, cellulose and nanocellulose can be easily modified due to their profusion of hydroxyl groups. Common strategies for producing cellulosic CO_2_ adsorbents include chemically modifying nanocellulose, incorporating inorganic particles into nanocellulose, and modifying nanocellulose with the addition of polymers [63].

An example of chemically modified nanocellulose is nanocellulose aerogel, which is created from the crosslinking and drying of nanocellulose [69]. Nanocellulose aerogel has a high surface area but must be chemically modified to achieve high CO_2_ selectivity. Liu et al. modified spherical cellulose nanofibril (CNF) hydrogel by introducing 3–5 wt% N-(2-aminoethyl)-3 aminopropylmethyldimethoxysilane in water at 80 °C or 90 °C for a duration of 10 h, followed by freeze drying [70]. The resulting hydrogel attained a CO_2_ adsorption capacity of 1.28–1.78 mmol/g at the higher temperature. This result was supported by a similar study conducted a year later by Zhang et al., who produced an N-(2-aminoethyl)-3 aminopropylmethyldimethoxysilane-modified spherical cellulose nanocrystal (CNC) aerogel with a CO_2_ adsorption capacity of 1.68 mmol/g [71]. Inorganic particles have also been employed to increase the CO_2_ adsorption of nanocellulose adsorbents. More specifically, Valencia et al. used silicalite-1 zeolite to modify a hybrid CNF-gelatin foam [72]. The resulting composite was able to adsorb up to 1.2 mmol CO_2_/g, which is comparable to the CO_2_ adsorption of pure silicalite-1. When the foam was further modified by incorporating a zeolitic imidazolate metal-organic framework (ZIF), the CO_2_ adsorption and selectivity over nitrogen were found to be improved, and this was attributed to the hierarchical porous structure of ZIF that can facilitate strong interaction with CO_2_ in the micropores [73].

**Figure 4:**
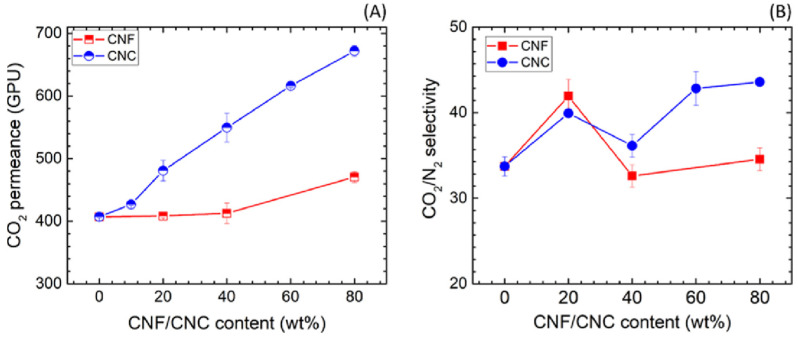
CO_2_ permeance (**A**) and CO_2_/N_2_ selectivity (**B**) of the hybrid nanocellulose/PVA membranes as a function of the nanocellulose content (Adapted from Dai et al. [76]).

Blending nanocellulose with hydrophilic polymers has also been found to improve a membrane’s CO_2_ permeability [74]. Venturi et al. incorporated 30% nanofibrilated cellulose (NFC) into a polyvinylamine membrane to increase its CO_2_ permeability and CO_2_/N_2_ selectivity [75]. The modified membrane had a CO_2_ permeability of 187 Barrer, a CO_2_/N_2_ selectivity of 100%, and a CO_2_/CH_4_ selectivity of 22% at 80% relative humidity. In another study, Dai et al. found that hybrid nanocellulose-80%/polyvinyl alcohol (PVA) membranes exhibit higher CO_2_ permeance if they are prepared with cellulose nanocrystals (CNC) rather than cellulose nanofibrils (CNF) [76]. As it can be seen in Figure 4, the 80 wt% CNC/PVA membrane had a 65% increase in CO_2_ permeance compared to the neat PVA membrane, while the 80 wt% CNF/PVA membrane only had a 15% increase in CO_2_ permeance with respect to the PVA membrane. Comparatively, the type of nanocellulose had a negligible effect on the hybrid membranes’ CO_2_/N_2_ selectivity. The 80 wt% CNC/PVA membrane was also able to maintain its high CO_2_ permeance and CO_2_/N_2_ selectivity for over a year.

**Figure 5:**
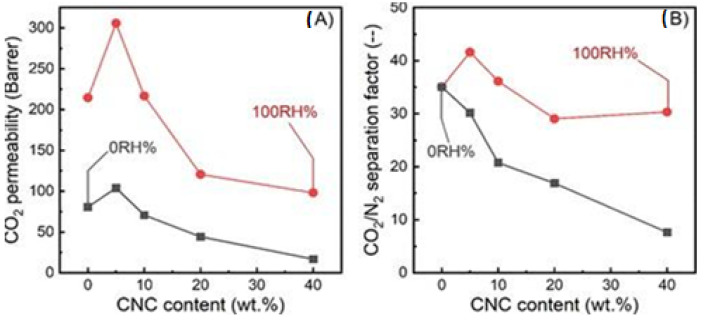
(**A**) CO_2_ permeability and (**B**) CO_2_/N_2_ selectivity of CNC/Pebax membranes under dry (0 RH%) and fully humid (100 RH%) conditions, respectively (Adapted from Dai et al. [74]).

In a more recent study, Dai et al. blended cellulose nanocrystals with Polyether-block-amide (Pebax 1657) to produce Pebax/CNC hybrid membranes [74]. The membranes were characterized by mixed-gas permeation tests under dry (relative humidity (RH) = 0%) and humid (RH = 100%) conditions. The results can be summarized in Figure 5. Under dry conditions, it was observed that 5 wt% CNC loading enhanced CO_2_ permeability by 29% (104.0 Barrer) compared to membranes with 0 wt% CNC loading. However, the CO_2_/N_2_ selectivity was not found to improve with the addition of CNC to the membrane. Under humid conditions, it was found that membranes with 5 wt% CNC loading increased CO_2_ permeability by 42% (305.7 Barrer) and CO_2_/N_2_ selectivity by 18% (41.6 separation factor) compared to membranes with 0 wt% CNC loading. Increasing humidity led to higher CO_2_ permeability and CO_2_/N_2_ selectivity, as can be seen in Figure 5. However, a common conclusion for both dry and humid conditions was that further increases in the CNC content above 5 wt% resulted in reduced CO_2_ permeability and CO_2_/N_2_ selectivity. This was attributed to the fact that as the CNC content increases, CNC tends to form a more oriented alignment, resulting in highly packed and complicated hierarchical structures that limit the gas diffusion and, thus, the transport through the CNC/Pebax membranes [74].

Table 3 summarizes the findings of the research works studied in this section.

### 3.3. Biomedical Applications

Another area of interest for nanocellulose is biomedicine. Many unique properties of nanocellulose, including crystallinity, high specific surface area, good rheological properties, ease of alignment, barrier properties, surface chemical reactivity, biocompatibility, and most notably, a lack of toxicity, render this material ideal for use in various biomedical applications, such as immobilization of enzymes, prevention of microbial growth, drug delivery, and virus removal [77,78,79,80,81,82,83]. More specifically, nanocellulose hydrogels produced from bacterial or plant cellulose nanofibrils were found to promote cell regeneration and can be applied to tissue engineering scaffolds [84]. Tissue scaffolds assist with wound dressing and cartilage repair that require low cytotoxicity and biocompatibility with the extracellular matrix, which is what nanocellulose hydrogels can provide [85,86]. In addition to its low toxicity, nanocellulose is favored for biosensors due to its biodegradable nature [67]. Biosensors are devices that measure and monitor diagnostic, environmental, safety, and security parameters [33,87].

Another biomedical application of nanocellulose membranes is in Surgicel^®^, which is a bio-absorbable hemostatic material employed in the prevention of surgery-derived adhesions [88]. Bacterial nanocellulose membranes were produced through electrochemical oxidation with the Tetramethylpiperidine-1-oxyl (TEMPO) radical to be applied to Surgicel^®^ for further improving its hemostatic performance [89]. This improvement was attributed to the enhanced oxidation degree, which increased from 4% to up to 15%. The in vivo biodegradability and biocompatibility of the resulting oxidized nanocellulose-based membranes were assessed through subcutaneous implantation of the membranes in rats and showed a highly biocompatible behavior, triggering only a mild inflammation process [89].

**Figure 6:**
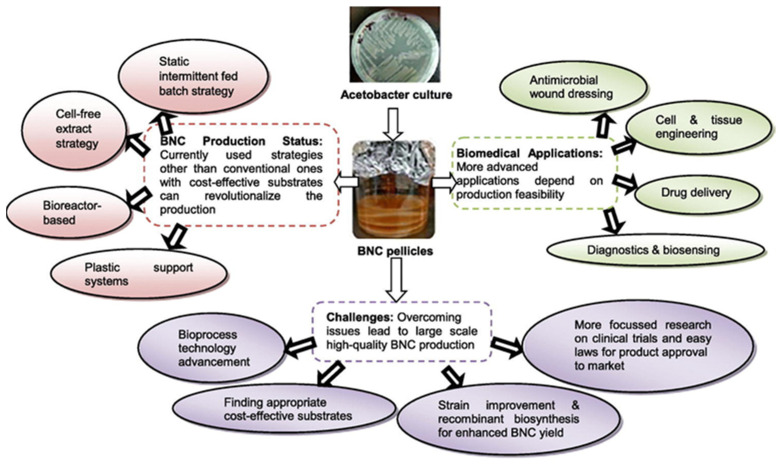
BNC production strategies, biomedical applications, and trends for overcoming challenges (Adapted from Sharma et al. [33]).

Figure 6 summarizes important biomedical applications of BNC, including biosensors and tissue engineering [33]. The realization of these applications depends on the production feasibility of BNC and nanocellulose in general. Therefore, Sharma et al. [33] address the BNC synthesis strategies in their study, suggesting the utilization of cost-effective substrates that may overcome the barriers associated with BNC production at large scale. These substrates can include agricultural wastes or wastewater rich in sugars from industrial effluents [33]. The challenges for the successful implementation of the nanocellulose biomedical applications are also made important in this study, including the tailoring of the cost of the substrates and the necessary legislation for product approval.

Nanocellulose hydrogels have also been employed as a medium for a gel-based blood type test [80]. Curvello et al. showed that traditional gel-based blood typing tests rely on microbeads, which do not always provide well-defined results [90]. This study found that gel columns containing at least 0.3 wt% 2,2,6,6-tetramethylpiperidine-1-oxyl (TEMPO)-oxidized CNFs were able to identify agglutinated and individual red blood cells (RBC) for forward blood typing [80]. TEMPO was chosen because TEMPO-oxidized cellulose can form nanofibers that have hydrophilic carboxylate groups [91]. For reverse blood typing, CNF can be crosslinked with hexamethylenediamine (HMDA) to achieve red blood cell agglutination and separation [90]. Compared to traditional materials used for blood typing tests, nanocellulose is inexpensive and sustainable. In addition to blood typing, nanocellulose hydrogels have shown promise as 3D cell cultures because they are able to accurately portray the extracellular matrix [92].

## 4. Future of Nanocellulose

Nanocellulose holds unique potential for a variety of applications that have already been addressed in this paper, including desalination, gas separation, automotive applications, and packaging. As such, new research should attempt to find novel uses for nanocellulose beyond pre-established applications. In addition to being investigated as a potential solution to water scarcity and global warming, this green nanomaterial may also pose a solution to the energy crisis; it can constitute an alternative option to fossil fuels by being employed as a sustainable and environmentally friendly material for renewable electronics [93]. Yang et al. posited that nanocellulose membranes can be used for osmotic energy harvesting [94]. Traditionally, osmotic energy harvesting technology has suffered from the required nanofluidic materials being too expensive to justify their practical application. However, Yang et al. found that inexpensive, yet effective membranes can be prepared by cross-linking CNF with 1,2,3,4-butanetetracarboxylic acid (BTCA) [94]. More research should be conducted to fully evaluate nanocellulose’s potential in renewable energy harvesting.

Numerous research studies related to food packaging have also been carried out. With plastics being the prominent material in modern packaging, an abundance of plastic pollution along coastlines has resulted from their use. Recently, nanocellulose has emerged as a potential solution for packaging materials. Since nanocellulose is harvested from plant fibers, it can naturally decompose over time, eliminating the issue of waste generation. Representative research works conducted in this area include the isolation of high crystalline nanocellulose from Mimosa pudica plant fibers for packaging applications [1], as well as applying nanocellulose as a starch-based packaging material for food [13]. While at its current stage of development, nanocellulose may not replace plastics entirely as the primary packaging option due to the profit-prioritizing business model of industries; however, it does help promote improvements to address modern issues in our society, such as improved gas barrier properties of the packaging materials. The potential of nanocellulose is seemingly endless. From acoustic materials [95] and cosmetics [96] to complex battery matrices [97] and optical materials [98], a wide range of applications can be implemented with this unique material.

## 5. Conclusions

Recent studies have shown that nanocellulose has a promising future as a nanomaterial in applications in numerous industrial fields. Advantageous properties, including biodegradability, non-toxicity, low density, thermal stability, long-lasting reinforcing capabilities, and high mechanical strength, have garnered interest in areas such as desalination wastewater treatment, gas separation, and biotechnologies. However, despite all the aforementioned qualities of nanocellulose, further studies are required in order to optimize its production [99] and valorization [100]. Specifically, the high cost, long processing time, and low yield of standard production processes prove to be challenges for this nanomaterial. The elevated cost is mainly attributed to the high energy consumption of the process. However, recent research has shown that these areas of concern can be addressed through the latest technologies. For instance, a drawback of nanocellulose has been its low thermal stability, but in a recent study by Chen et al., a highly thermally stable nanocellulose-based flexible material was developed that can be utilized in electronics [101].

In conclusion, nanocellulose holds limitless potential in both conventional and unconventional applications. Generally, researchers have focused on nanocellulose’s uses in the preparation of chemicals or their handling, such as in separations, desalination, and packaging. But nanocellulose may also have uses in the natural and life sciences, including dermal care applications. Chantereau et al. have shown that bacterial nanocellulose membranes loaded with vitamin-based ionic liquids are ideal candidates for skin care applications due to their high thermal stability and increased solubility [102]. With further advancements, it may even have recreational uses, such as a material for musical instruments [95]. The future of nanocellulose should not be limited to one area of study but instead expanded to reach as many as possible.

## Figures and Tables

**Table 1:** Comparison of structure parameters of different types of nanocellulose. (Adapted from Huo et al. [31]).

	Structural Properties			Mechanical Properties	
Nanocellulose Type	Length (nm)	Diameter (nm)	Crystallinity (%)	Young’s Modulus (GPa)	Tensile Strength (GPa)
CNCs	100–500	3–50	~90	50–140	8–10
CNFs	≥10^3^	3–60	50–90	50–160	0.8–1
BC	≥10^3^	20–100	84–89	78	0.2–2

**Table 2:** Summary of the performance of nanocellulose-based materials tested for water treatment applications.

Nanocellulose-Based Membrane Material	Summary of Results	Ref.
Polydopamine-modified Cellulose nanocrystals (CNCs)	✓ Pure water permeability of 128.4 L m^−2^ h^−1^ (LMH)/bar ✓ Congo red rejection of 99.91% ✓ Salt permeation of 99.33%	[44]
Reduced graphene oxide (RGO)/cellulose nanofibrils (CNFs)	Varying rGO loading at a fixed 1:1 ratio of rGO to CNF and testing of MO, RhB, AF, BB and RB dyes: Pure water permeance of 37.2 LMH/bar. Only BB and RB exhibited more than 90% rejection in all studied membranes.	[45]
Negatively charged carboxylated CNF with trimethylolpropane-tris-(2-methyl-1-aziridine) propionate and graphene oxide	Excellent adsorbent of numerous cations of heavy metals, including Pb^2+^, Cd^2+^ and Cu^2+^	[48]
Tetramethylpiperidine-1-oxyl (TEMPO)-oxidized nanocellulose	Adsorption capabilities for divalent cations	[50]
CNF modified by nitro-oxidation	Adsorption of Cd^2+^ was optimal at pH 7 and decreased in acidic and basic conditions	[51]
Polydopamine (PDA) particles and bacterial nanocellulose	Salt rejection > 99.9% 1.0 kg m^−2^ h^−1^ permeate flux	[54]
Nanocellulose acetate (NCA)	Salt rejection of 97.4% Water flux of 2.2 L/m^2^ h	[55]
Microporous network of cellulose composite	High evaporation rate and salt resistance within evaporator	[57]
BNC loaded with a high concentration of polydopamine (PDA)	Solar steam generation efficiency of 78% within evaporator	[58]

**Table 3:** Summary of the performance of nanocellulose-based materials tested for water treatment applications.

Nanocellulose-Based Membrane Material	Results	Ref.
Modified spherical cellulose nanofibril (CNF) hydrogel	CO_2_ adsorption capacity of 1.28–1.78 mmol/g at high temperature	[70]
N-(2-aminoethyl)-3 aminopropylmethyldimethoxysilane modified spherical cellulose nanocrystal (CNC) aerogel	CO_2_ adsorption capacity of 1.68 mmol/g	[71]
Silicalite-1 zeolite modified hybrid CNF-gelatin foam	Adsorption up to 1.2 mmol CO_2_/g	[72]
Hybrid CNF-gelatin foam with a zeolitic imidazolate metal-organic framework (ZIF)	Greater CO_2_ adsorption and selectivity over nitrogen	[73]
Pebax/CNC hybrid membranes	Under dry conditions, 5 wt% CNC loading resulted in: Enhancement of CO_2_ permeability by 29% (104.0 Barrer) No improvement in CO_2_/N_2_ selectivity compared to membranes with 0 wt% CNC loading.	[74]
Pebax/CNC hybrid membranes	Under humid conditions, 5 wt% CNC loading led to improvement of: CO_2_ permeability by 42% (305.7 Barrer) CO_2_/N_2_ selectivity by 18% (41.6 separation factor) compared to membranes with 0 wt% CNC loading. Increasing humidity resulted in higher CO_2_ permeability and CO_2_/N_2_ selectivity.	[74]
30% nano-fibrilated cellulose (NFC) into a polyvinylamine membrane	CO_2_ permeability of 187 Barrer, CO_2_/N_2_ selectivity of 100%, and CO_2_/CH_4_ selectivity of 22% at 80% relative humidity	[75]
Hybrid nanocellulose (80%)/polyvinyl alcohol (PVA) membranes	80 wt% CNC/PVA membranes exhibited a 65% increase in CO_2_ permeance compared to the pure PVA membrane. (80 wt% CNF/PVA membrane had a 15% increase in CO_2_ permeance with respect to the pure PVA membrane.)	[76]

## Data Availability

Not applicable.
